# Reduction of Maternal Mortality with Highly Active Antiretroviral Therapy in a Large Cohort of HIV-Infected Pregnant Women in Malawi and Mozambique

**DOI:** 10.1371/journal.pone.0071653

**Published:** 2013-08-19

**Authors:** Giuseppe Liotta, Sandro Mancinelli, Karin Nielsen-Saines, E. Gennaro, Paola Scarcella, Nurja Abdul Magid, Paola Germano, Haswell Jere, Gianni Guidotti, Ersilia Buonomo, Fausto Ciccacci, Leonardo Palombi, Maria Cristina Marazzi

**Affiliations:** 1 Department of Biomedicine and Prevention, University of Tor Vergata, Rome, Italy; 2 Department of Pediatrics, David Geffen UCLA School of Medicine, Los Angeles, California, United States of America; 3 Department of Public Health, University G. D’Annunzio, Chieti, Italy; 4 DREAM Program Department, Community of Sant’Egidio, Maputo, Mozambique; 5 DREAM Program Department, Community of Sant’Egidio, Rome, Italy; 6 DREAM Program Department, Community of Sant’Egidio, Blantyre, Malawi; 7 Department of Infectious Diseases, National Institute of Infectious Diseases, Rome, Italy; 8 Department of Community Health, Lumsa University, Rome, Italy; 9 Department of Infectious Diseases, University La Sapienza, Rome, Italy; University of North Carolina School of Medicine, United States of America

## Abstract

**Background:**

HIV infection is a major contributor to maternal mortality in resource-limited settings. The Drug Resource Enhancement Against AIDS and Malnutrition Programme has been promoting HAART use during pregnancy and postpartum for Prevention-of-mother-to-child-HIV transmission (PMTCT) irrespective of maternal CD4 cell counts since 2002.

**Methods:**

Records for all HIV+ pregnancies followed in Mozambique and Malawi from 6/2002 to 6/2010 were reviewed. The cohort was comprised by pregnancies where women were referred for PMTCT and started HAART during prenatal care (n = 8172, group 1) and pregnancies where women were referred on established HAART (n = 1978, group 2).

**Results:**

10,150 pregnancies were followed. Median (IQR) baseline values were age 26 years (IQR:23–30), CD4 count 392 cells/mm^3^ (IQR:258–563), Viral Load log_10_ 3.9 (IQR:3.2–4.4), BMI 23.4 (IQR:21.5–25.7), Hemoglobin 10.0 (IQR: 9.0–11.0). 101 maternal deaths (0.99%) occurred during pregnancy to 6 weeks postpartum: 87 (1.1%) in group 1 and 14 (0.7%) in group 2. Mortality was 1.3% in women with <than 350 CD4 cells/mm^3^ and 0.7% in women with greater than 350 CD4s cells/mm^3^ [OR = 1.9 (CL 1.3–2.9) *p* = 0.001]. Mortality was higher in patients with shorter antenatal HAART: 22/991 (2.2%) if less than 30 days and 79/9159 (0.9%) if 31 days or greater [OR = 2.6 (CL 1.6–4.2) *p*<0.001]. By multivariate analysis, shorter antenatal HAART (*p*<0.001), baseline values for CD4 cell count (*p* = 0.012), hemoglobin (*p* = 0.02), and BMI (*p*<0.001) were associated with mortality. Four years later, survival was 92% for women with shorter antenatal HAART and 98% for women on established therapy prior to pregnancy, p = 0.001.

**Conclusions:**

Antiretrovirals for PMTCT purposes have significant impact on maternal mortality as do CD4 counts and nutritional status. In resource-limited settings, PMTCT programs should provide universal HAART to all HIV+ pregnant women given its impact in prevention of maternal death.

## Introduction

Maternal death classically encompasses the period of pregnancy until 42 days after the termination of gestation, and is attributable to causes related or aggravated by pregnancy or its management. Pregnancy related-deaths, however, are defined as maternal deaths occurring during the same time period irrespective of cause. Late maternal deaths encompass the period between 42 days post pregnancy up to one year within termination of gestation and include deaths from direct or indirect obstetric causes. In resource limited settings, maternal mortality is approximately fifteen times as high as that of developed nations [1]. In 2010, for example, 287,000 maternal deaths were reported globally, of which 56% occurred in Sub-Saharan Africa, where the average Maternal Mortality Rate (MMR) was 500 deaths per 100,000 live births with 10% of deaths occurring as a direct consequence of HIV infection [1]. Conversely, of 19,000 maternal deaths reportedly attributed to HIV worldwide, 91% occurred in Sub-Saharan Africa [1]. HIV infection is more frequently an indirect (or non-obstetrical) cause of maternal death, which is difficult to quantify in this setting. When a pregnant woman dies because of HIV-related complications, the cause of death is generally classified as AIDS-related under these circumstances. Similarly pregnancy is a condition which tends to worsen HIV infection and its co-morbidities. Therefore, due to these differences in categorization and timing of maternal mortality, it is difficult for surveillance programs to gauge the impact of HIV on maternal mortality and also the impact of pregnancy on HIV-related deaths. Undoubtedly, however, HIV-infection is a major contributor to maternal mortality in resource-limited settings. Countries which originally noted an acceleration in maternal mortality because of HIV pandemic in the last decade have seen a decline in maternal mortality in recent years with the scaling up of antiretroviral treatment to pregnant women [2].

The Drug Resource Enhancement Against AIDS and Malnutrition Program (DREAM) run by the Community of Sant’Egidio, a large Christian movement with a strong presence in Africa, provides comprehensive HIV-care in several Sub-Saharan African countries. The program has routinely provided Highly Active Antiretroviral Therapy (HAART) to all HIV-infected pregnant women for Prevention of Mother-To-Child HIV transmission (PMTCT) since 2002, irrespective of CD4 cell count, during prenatal care and in the postpartum period during breastfeeding. This broad intervention was made possible by the development of country-specific treatment protocols, through partnership agreements with the respective Ministries of Health. Over the years improved pregnancy outcomes have been observed in patients receiving triple ART in the program. We previously reported a significant association between extended antenatal HAART and reduced maternal mortality and improved pregnancy outcomes (like reduction of abortion/stillbirth, prematurity and low birth weight rates) in a cohort of 3000 HIV-infected pregnant women followed in the program [3]. In the present analysis, we evaluate maternal mortality, antiretroviral uptake, laboratory parameters and loss to follow-up in 2 distinct pregnant populations: HIV-infected women initiating HAART during pregnancy for PMTCT purposes and women on established antiretroviral therapy who became pregnant while on treatment. Risk factors for maternal mortality during pregnancy up to 6 weeks postpartum were assessed in both populations, as well as risk factors for mortality beyond the immediate postpartum period, for up to 4 years.

## Materials and Methods

### Study Design

Retrospective observational cohort study.

### Study Population

Data from HIV-1-infected pregnant women accessing any of the 16 DREAM centers for prenatal care in Malawi (2006–2009) and Mozambique (2002–2010) was evaluated. Inclusion criteria included: (1) Prenatal care at participating DREAM centers; (2) Availability of medical records in the database (3) Maternal written informed consent for participation in the program. Data were analyzed in a blinded fashion with removal of patient identifiers. Patients were categorized in 2 groups: (1) Women initiating triple ART during prenatal care and (2) Women initiating ART for their own health prior to pregnancy (on established ART when pregnancy occurred). Criteria for initiating ART prior to pregnancy were based on WHO clinical or immunologic criteria, including CD4 cell counts of less than 350 cells/mm^3^ or clinical findings. The standard HAART regimen was nevirapine (NVP)-based, although protease-inhibitor based regimens were also used in a subset of patients. Women identified in pregnancy were offered NVP- based triple ART at 14 weeks (if required for their own health) or from 25 weeks of gestation until 6 months postpartum if used for PMTCT purposes. The study was approved by institutional review boards and regulatory institutions in Italy (IRB of the Tor Vergata University School of Medicine), Mozambique (Comite de Etica do Ministerio de Saude de Moçambique- Mozambique Ministry of Health IRB) and Malawi (Ministry of Health of Malawi, Ethics Committee). The study received an exemption from informed consent as all data was analyzed anonymously and retrospectively.

### Methods

Electronic data were pooled from DREAM centers in Mozambique and Malawi, as the program uses electronic files for monitoring all clinical visits including laboratory parameters and nutritional status [4]. All files available for mothers followed in PMTCT programs from June 2002 to June 2010 were reviewed. Data collected included maternal demographics, obstetrical and medical history, gestational age, body mass index, laboratory parameters including HIV-1 virus load, T cell subsets, hemoglobin and transaminases, antiretroviral history, duration, mortality and loss to follow-up. Clinical centers use electronic medical records linked to medical, nutritional, and laboratory data.

### Study Outcomes

Maternal mortality was defined as death during pregnancy, at delivery or within 42 days after delivery. Mortality beyond the immediate postpartum period was defined as death between 42 days following termination of pregnancy up to 4 years post-delivery. Antiretroviral uptake and loss to follow-up were also assessed. Statistical Analysis: SPSS v. Win 19.0.1 was used for data analysis. Data were censored at the time of death, or at the last patient visit before loss to follow-up, or on 6/30/2010. Median values were provided with corresponding interquartile range (IQR) and mean values with standard deviation (SD). Ninety-five percent confidence intervals were calculated and Pearson *Χ^2^* test was used for assessment of potential differences in mortality, antiretroviral uptake, loss to follow-up and laboratory parameters between groups. To assess the statistical significance of comparison between means an univariate ANOVA procedure was carried out using the Tamhane test assuming not equal variances (p-level = 0.05). The Multivariate Cox Regression Hazard risk analysis was used to describe survival according to pre-delivery length of HAART and adjusted for baseline values of key variables including viral load, CD4 count, BMI and hemoglobin.

## Results

Over 8 years, 10,150 pregnancies in 8661 HIV-infected pregnant women were followed through the program in any of 16 participating centers in Mozambique or Malawi. A total of 9261 infants were delivered (8816 singletons, 436 twins and 9 triplets), of which 8959 (97%) were live births. Overall, the cohort was comprised by a relatively healthy group of women, with a median age of 26 years (IQR: 23–30) and a median CD4 count at baseline of 392 cells/mm^3^ (IQR: 258–563). All patients had detectable HIV viral loads at baseline prior to initiation of HAART (log_10_ 3.9; IQR : 3.2–4.4) with a mean body mass index (BMI) of 23.4 (IQR: 21.5–25.7) and a median hemoglobin value of 10.0 g/dL (IQR: 9.0–11.0). Within the cohort, for 8172 pregnancies, women initiated HAART during prenatal care and in 1978 pregnancies, women were on established HAART when presenting for prenatal care. Baseline parameters prior to initiation of HAART for both groups are presented in Table 1. Women in the cohort who originally initiated triple ART during pregnancy had a mean time of triple ART of 2.3 years at the time of analysis while the group who initiated treatment for their own health prior to pregnancy had a mean time of 4 years on therapy. The groups were statistically different in terms of pre-HAART CD4 cell counts (higher in the PMTCT group), pre-HAART virus load (higher in the ART treatment group), pre-HAART BMI (higher in the PMTCT group), and loss to follow-up (higher in the PMTCT group). Parity was different between the 2 groups, as most pregnancies in the PMTCT group were first gestations (83%), as compared to 44% first pregnancies among women on established ART, as seen in Table 1.

**Table 1:** Baseline parameters of cohort.

Baseline parameters prior to initiation of HAART	Group 1 N = 8172pregnancies Mean (SD)	Group 2 N = 1978 pregnancies Mean (SD)	*p*
Duration of HAART prior to delivery (days)	83 (46)	766 (419)	<0.001
Total Observation Time (days)	832 (586)	1459 (621)	<0.001
CD4 cell count (cells/mm^3^) (Missing, n = 66)	431 (275)	354 (366)	<0.001
Viral load (cps/ml) (Missing, n = 290)	3.6 (1.3)	3.8 (1.5)	<0.001
Body Mass Index (Missing n = 44)	23.8 (3.6)	22.0 (3.8)	<0.001
Hemoglobin (Missing, n = 32)	9.9 (3.4)	10.1 (1.9)	0.26
Loss to follow up	9.8%	3.9% (before delivery)	<0.001
Parity			
First pregnancies (N = 7610)	6748 (83%)	862 (44%)	
Second pregnancies (N = 1089)	352 (4%)	737 (37%)	
Third pregnancies (N = 129)	33 (0.4%)	96 (5%)	
Fourth pregnancies (N = 8)	1 (0.1%)	7 (0.4)	
Unknown (N = 1314)	1038 (13%)	276 (14%)	

Mortality within 42 days post delivery occurred in 101 patients (0.99%). It was higher in the PMTCT group (87/8158, 1.1%) versus the ART for treatment purposes group (14/1992, 0.7%) but this finding was not statistically significant (p = 0.17). As expected, maternal mortality was lower in women with higher CD4 cell counts at baseline (1.3% in women with <350 CD4 cells/mm^3^ and 0.7% in women with CD4 cell counts ≥350 cells/mm^3^) (OR = 1.9 (CL 1.3–2.9) *p* = 0.001). Mortality was also higher in patients with shorter duration of antenatal HAART: 22 deaths among 991 women (2.2%) with less than 30 days of antenatal HAART and 79 deaths among 9159 women (0.9%) with 31 days or greater of antenatal HAART (OR = 2.6 (CL 1.6–4.2) (*p*<0.001). Mortality rates within our cohort paralleled the maternal mortality observed in the general population (Mozambique MMR of 520 (0.52%) and Malawi MMR of 1100 (1.1%) in 2009) [1]. Table 2 demonstrates adverse pregnancy outcomes including death. Although abandonment of care was more frequent in the PMTCT group (7.8%) as opposed to the group of women on established ART (5.4%), (p<0.001), fetal loss (abortion/stillbirth) was slightly more prevalent among women on established therapy (6.5%) than women initiating ART for PMTCT (4.4%), p<0.001). As seen in Table 3, parameters associated with short term maternal mortality on multivariate Cox regression hazards risk analysis included shorter antenatal HAART exposure (*p*<0.001), baseline CD4 cell count (*p* = 0.021), baseline hemoglobin levels (*p* = 0.005), and baseline body mass index (BMI) (*p*<0.001).

**Table 2:** Adverse pregnancy outcomes.

	Group 1 N = 8172pregnancies	Group 2 N = 1978pregnancies	N = 10150
Maternal Mortality	1.1%	0.7%	0.17
Abandonment of care post-delivery	636 (7.8%)	107 (5.4%)	743 (7.3%)
Abortion	126 (1.5%)	68 (3.4%)	194 (1.9%)
Stillbirth	241 (2.9%)	61 (3.1%)	302 (3.0%)
Unknown fetal outcome	35 (0.4%)	40 (2%)	75 (0.7%)
Total adverse/unknown outcomes	1038 (12.7%)	276 (14%)	1314 (12.9%)

**Table 3:** Multivariate Cox Regression Hazard risk analysis.

Outcome variable:Maternal Death	p	RR	IC 95.0%	
Pre-delivery shorter ART exposure	<0.001	3.035	1.802	5.114
Baseline BMI	<0.001	.602	.453	.801
Baseline Hb	0.005	.660	.493	.882
Baseline Viral Load	0.440	1.134	.824	1.562
Baseline CD4 count	0.021	.884	.796	.982

BMI: <18.5, 18.5–20.0, >20.0;

Hb: <8.0 gr/100 mL, 8.0–10.0, >10.0;

Viral Load: <4.0 c_Log_/ml, 4.0–5.0 c_Log_/ml, >5.0 c_Log_/ml;

CD4 count: <100 cell/mL, 100–200, 200–300, 300–400, >400.

**Figure 1:**
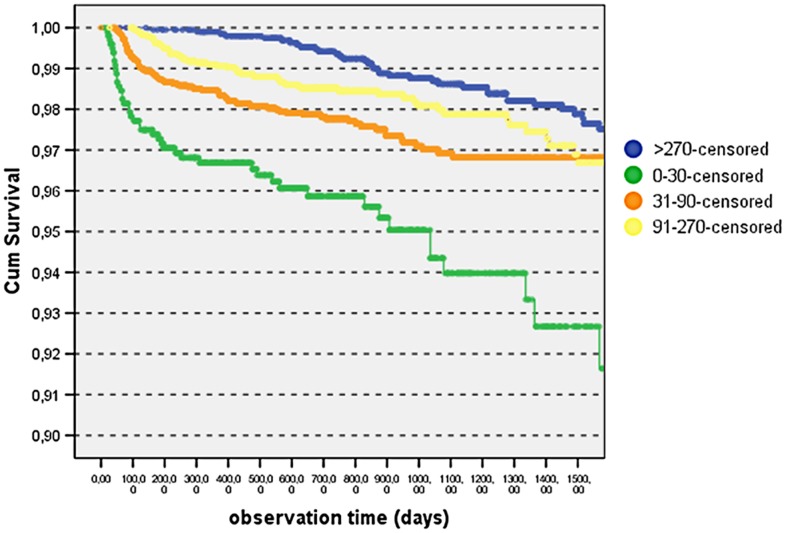
Kaplan-Meier maternal survival analysis according to length of pre-delivery HAART.

Among the 101 women who died within 42 days post-delivery, 41 died giving birth. This included 12 women with less than 31 days of antenatal HAART. Three deaths occurred in 482 Cesarean deliveries (0.6%), one death occurred among 116 deliveries reported as complicated by fetal dystocia (0.9%) and 37 deaths occurred among 8125 deliveries reported as normal, vaginal, eutocic deliveries (0.5%). Four additional deaths were associated with abortion (n = 1) or stillbirth (n = 3). Fifty-three deaths were reported among women who abandoned the program post-delivery, 9 women in this group (18%) had received less than 30 days of antenatal HAART. For 3 additional women who received less than 30 days of antenatal HAART no additional details regarding circumstances of death or fetal outcomes were available. Table 4 demonstrates stratification of the cohort by duration of antenatal HAART exposure and corresponding mortality, both short term and long term. Women receiving the shortest duration of antenatal HAART had the highest mortality at 2.2% while women receiving antiretrovirals for their own health with more advanced disease at baseline, had a mortality of 0.7%. Therefore, the short term mortality of women receiving less than a month of HAART prior to delivery (2.2%) was double that of women receiving from at least a month to 3 months of antenatal HAART (1.1%), and three-fold as high as that of women who had received more than 3 months of HAART prior to delivery (0.6%), p<0.0001 by Chi-square analysis. Interestingly, long term maternal mortality, ranging from 43 days post-partum to up to 4 years was also associated with duration of antenatal exposure to ART. Long term mortality among women who received less than a month of HAART before delivery was over 2-fold higher (4.5%) than that of women who received 3 months or more of antenatal HAART (1.8%), p<0.0001, as seen in Table 4. The table also reports incidence as events per 100 person/years which confirms the previously described results. The Kaplan-Meier survival analysis in Figure 1 demonstrates that the survival of women who received the shorter course of antenatal HAART was slightly less than 92% at four years, whereas the survival of women on established therapy prior to pregnancy (>270 days of antenatal HAART) bordered 98%, p = 0.001.

**Table 4:** Short term and long term maternal mortality according to duration of antenatal HAART (Incidence per 100 PY).

Short term Maternal Mortality						
Pre-delivery HAART (days)	N	Deaths	%	Deaths per 100 P/Y (mean)	DS	p
0–30	991	22	2.2	3.4	23.6	<0.001
31–90	3899	44	1.1	1.6	16.0	
91–270	3282	21	0.6	0.8	10.2	
>270	1978	14	0.7	0.9	11.3	
Totale	10150	101	1.0	1.4	14.6	
**Long term Maternal Mortality**						
**Pre-delivery HAART (days)**	**N**	**Deaths**	**%**	**Deaths per 100 P/Y (mean)**	**DS**	**p**
0–30	991	45	4.5	5.1	26.2	<0.001
31–90	3899	92	2.4	2.5	17.8	
91–270	3282	58	1.8	1.5	12.8	
>270	1978	39	2.0	1.7	14.0	
Totale	10150	234	2.3	2.3	16.7	

*p*-values are for the overall sample and refer to person-year incidence.

Strata-specific comparisons were performed: for short term mortality all comparisons were statistically significant except for 91–270 strata against the >270 strata; for long term mortality only the first strata (0–30 days) showed statistically significant differences.

**Figure 2:**
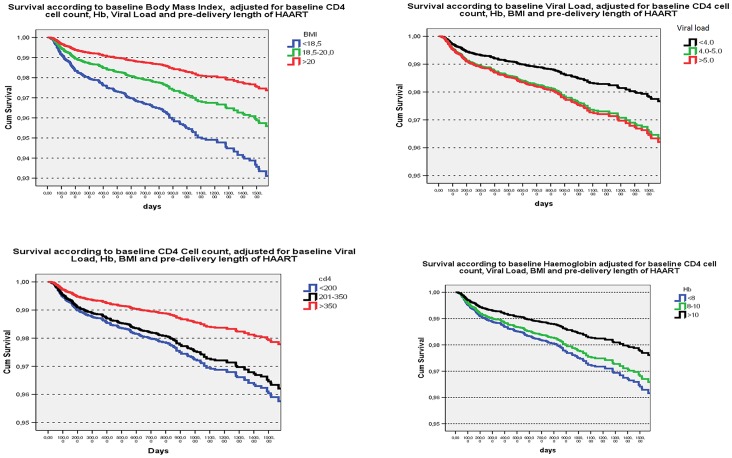
Kaplan-Meier maternal survival analysis according to baseline parameters: BMI, HIV-1 viral load, CD4 cell count and hemoglobin values.

The major factors associated with four year mortality by Cox regression analysis are shown in Table 5. A shorter course of no more than 30 days of triple ART prior to delivery, (HR: 5.5, *p*<0.0001), a lower virus load less than 4 C_Log_/ml (Protective HR: 0.6, *p* = 0.025), a CD4 cell count of less than 200 cells at baseline (HR: 1.9, p<0.0001) were associated with mortality risk. A baseline BMI of less than 20 and a hemoglobin baseline value of less than 10 g/DL were also significantly associated with mortality risk as shown in the table. These findings are illustrated in Figure 2, which demonstrates individual Kaplan-Meier survival analyses stratified by baseline CD4 cell count, baseline HIV-1 virus load, baseline hemoglobin levels and baseline BMI values.

**Table 5:** Four year mortality risk following delivery: Cox regression analysis.

	*p*- value	HR	95.0% CI for HR	
HAART >270 (reference)		1.0	Lower	Upper
0–30 days	<0.0001	5.5	3.5	8.7
31–90	<0.0001	2.8	1.9	4.3
91–270	0.003	1.9	1.2	2.8
Viral Load >5 Log (reference)		1.0		
<4 C_Log_/ml	0.025	0.6	0.3	0.9
4.0–5.0	0.857	0.9	0.6	1.4
* (Missing, n = 290)*				
CD4>350		1.0		
<200 Cell/microL	<0.0001	1.9	1.3	2.7
200–350	0.001	1.7	1.2	2.3
* (Missing, n = 66)*				
BMI >20.0 (reference)		1.0		
<18.5	<0.0001	2.7	1.8	4.0
18.5–20.0	0.012	1.7	1.1	2.5
* (Missing n = 44)*				
Hb >10.0 (reference)	0.019	1.0		
8.0–10.0 gr/100 cc	0.017	1.6	1.1	2.4
<8.0	0.016	1.4	1.1	1.9
* (Missing, n = 32)*				

## Discussion

In resource limited settings, improvements in maternal mortality have occurred in the last two decades for a number of reasons, despite the HIV-epidemic. These include an increase in the number of skilled birth attendants present at delivery from 55 to 65% from 1990 to 2009 [5], at least one visit during pregnancy with skilled health personnel, which saw an increase from 64 to 81% over the same period [5], lower pregnancy rates overall [5], and the roll-out of antiretrovirals in Sub-Saharan Africa, from less than 10% in 2000 to 55% by 2010 [2]. Countries like Botswana, South Africa, Lesotho, Swaziland and Namibia which saw an acceleration in maternal mortality in the past decade because of HIV infection, observed a decline in maternal mortality ratios in recent years because of the wider availability of HAART during prenatal care, following the attainment of 80% antiretroviral coverage to all pregnant women living with HIV as recommended by the 2001 UN General Assembly Special Session (UNGASS) [1], [2]. Data from our group and also others has demonstrated an early mortality following HAART initiation when antiretrovirals are started at very low CD4 cell count thresholds [6], [7], [8]. In such situations HAART was initiated too late in the course of disease, without ample time for reversal of immunodeficiency before a fatal outcome. When antiretrovirals are initiated during pregnancy for PMTCT purposes at higher CD4 cell count levels this risk of early mortality following treatment initiation because of very advanced HIV disease is circumvented. In our cohort, women on established ART who became pregnant did not have a higher mortality rate (0.7%) than women initiating ART during prenatal care (1.1%), despite more advanced HIV disease in the treatment-experienced group (who had lower CD4 cell counts and lower BMI at baseline). A longer duration of HAART exposure prior to delivery was associated with a significant survival benefit, regardless of baseline status, as demonstrated in our Kaplan-Meier analysis. Women on established ART were also less likely to be lost to follow-up, as compared to women who engaged into care for PMTCT purposes.

In Sub-Saharan Africa, maternal mortality and HIV disease are converging epidemics [9]. When one considers that the average MMR of the region is 500 deaths per 100,000 births [1], the risk of death for a pregnant woman in this setting is 1 in 39. If HIV infection is taken into account, the risk of death during pregnancy is double, or 1 in 20 if women are not on ART [10]. This proportion can reach extremely elevated rates, 10 to 13-fold higher among HIV-infected women as compared to uninfected, depending on the immune status of the HIV population under consideration [11], [12], in the absence of antenatal HAART. Our data, based on the observation of 10,150 pregnancies of HIV-infected women over an eight year period, clearly demonstrates the impact of antenatal HAART on reduction of overall maternal mortality. In our multivariate analysis, a reduced course of less than a month of antenatal HAART increased the risk of death three-fold. This parameter was the most robust in our analysis, followed by baseline BMI, hemoglobin values or CD4 cell counts.

Deaths in HIV-infected women are generally attributed to advancing immunodeficiency and worsening co-morbidities such as anemia, malnutrition, malaria or tuberculosis. It appears, however, that HIV also increases the risk of death due to obstetrical complications, including hemorrhage, miscarriage or sepsis [11], [13], [14]. Pregnancy is a vulnerable immunologic state where women are more prone to acquiring HIV infection [15]. As such, pregnancy is also associated with a higher likelihood of development of other viral respiratory infections, bacterial pneumonia and TB, all exacerbated by HIV [16], [17]. In our study, among the 101 women who died within 42 days of termination of pregnancy, 41 died in the process of giving birth, presumably of obstetrical complications, while 4 others died due to complications leading to concurrent fetal demise during the labor process. The additional 56 women who died in the immediate postpartum period did not return for care at our centers. While their deaths were ascertained through home visits made by our staff, cause of death was not recorded. We can conclude from our findings, nevertheless that at least 45% of the short term maternal mortality cases resulted from obstetrical complications. As cause of death was not consistently recorded for long term mortality cases, we cannot report on the exact cause of death for the 234 women who died as of 43 days following delivery. In our general patient population, the most frequently reported causes of death in descending order are malaria, anemia and tuberculosis [6]. Our group previously demonstrated that in a cohort of 3000 pregnant patients, receipt of HAART for at least 3 months antenatally was associated with a mortality rate of 0.7% [3]. Our present data originates from a sample size of 10,150 pregnancies and confirms a very significant survival benefit when HAART is initiated early in pregnancy. The highest survival benefit was clearly associated with extended duration of antenatal HAART, with patients on established therapy faring the best in terms of survival. The use of extended HAART prior to delivery was not only associated with improvement of immediate maternal mortality but carried a survival benefit still visible four years later. Additional risk factors for long term maternal mortality were not surprising, and included parameters associated with advanced disease at baseline, including lower CD4 cell counts, anemia and malnutrition. Other adverse outcomes including abandonment of care or fetal demise occurred in 13% of pregnancies. Women on established treatment prior to pregnancy were less likely to abandon care (5.4%) than women initiating ART during pregnancy (7.8%), which is consistent with our clinical experience.

One potential study limitation was that deaths in the first trimester of pregnancy among mothers who were not receiving HAART at conception might not have been readily identified. However this phenomenon is also true for HIV negative mothers in many developing country settings as it is uncommon for pregnancies to be recognized before the third month of gestation. Therefore this would be likely a problem prevalent in all populations. Most maternal deaths in the first trimester tend to be due to ectopic pregnancies which accounts for less than 10% of direct causes of maternal death and less than 5% of the cumulative number of direct and indirect causes of maternal deaths. Therefore the contribution of early trimester deaths to the maternal death rate is likely not to have been as significant. Another potential study limitation was that IRIS was not accessed as a cause of death. Nevertheless the proportion of patients with less than 200 CD4 cell counts was clearly less within the group of women who started treatment during pregnancy as compared to women who initiated treatment before pregnancy. In our cohort, women who started treatment before pregnancy were in worse immunologic condition with lower CD4 cell counts at baseline (data not shown). Based on these observations it is unlikely that IRIS would have played a role in the increased mortality rate observed in women with less than 30 days of pre-delivery HAART.

While fetal demise was slightly more prevalent among women on established ART (6.5%) than women who initiated ART during pregnancy for PMTCT (4.4%), this finding needs to be interpreted with caution, as women tend to present relatively late during pregnancy for care in our setting. Early trimester miscarriages would not likely be identified in the PMTCT cohort as they would happen before women sought medical care, while in the other group routinely followed in care, fetal demise would be more likely to be identified. Another explanation is that women with more advanced disease would be more prone to fetal demise, although this hypothesis cannot be ascertained from our data.

## Conclusions

The geographic clustering of the burden of disease in Sub-Saharan Africa is responsible for superimposing epidemics of malaria, TB, enteric diseases, HIV and malnutrition. If one takes into account that approximately 1,000,000 pregnancies per year are complicated by malaria and HIV co-infection [18], and that the rate of TB/HIV co-infection is approximately 30% [18], there is the potential for 300,000 pregnancies per year complicated by triple infection. Maternal nutritional status, another predictor of adverse pregnancy outcomes, is compounded by AIDS and a low BMI, which is shown by our data to be associated with mortality risk. In both cohorts evaluated in the present study, provision of triple ART in pregnancy was associated with a significant survival benefit which rendered maternal mortality in HIV+ women equivalent or lower to population country-wide statistics (1%) and significantly lower than the mortality of untreated HIV+ mothers. In conclusion, our data supports the need for universal access to antiretrovirals during pregnancy, as antiretrovirals prevent mortality, mothers included.
