# Correction: Reduction of Maternal Mortality with Highly Active Antiretroviral Therapy in a Large Cohort of HIV-Infected Pregnant Women in Malawi and Mozambique

**DOI:** 10.1371/annotation/9ab4708e-650e-411a-b72f-bb85e0845469

**Published:** 2013-08-28

**Authors:** Giuseppe Liotta, Sandro Mancinelli, Karin Nielsen-Saines, E. Gennaro, Paola Scarcella, Nurja Abdul Magid, Paola Germano, Haswell Jere, Gianni Guidotti, Ersilia Buonomo, Fausto Ciccacci, Leonardo Palombi, Maria Cristina Marazzi

In the author byline, name of the fourth and ninth authors were incorrect.

The correct name is: Elisabetta Gennaro.

The correct name of the ninth author is: Giovanni Guidotti.

The ninth author was also affiliated with the incorrect institution. The correct institution is:

DREAM Program Department, Community of Sant’Egidio, Blantyre, Malawi 

